# Glucocorticoid mediated inhibition of LKB1 mutant non-small cell lung cancers

**DOI:** 10.3389/fonc.2023.1025443

**Published:** 2023-03-23

**Authors:** Kenneth E. Huffman, Long Shan Li, Ryan Carstens, Hyunsil Park, Luc Girard, Kimberley Avila, Shuguang Wei, Rahul Kollipara, Brenda Timmons, Jessica Sudderth, Nawal Bendris, Jiyeon Kim, Pamela Villalobos, Junya Fujimoto, Sandra Schmid, Ralph J. Deberardinis, Ignacio Wistuba, John Heymach, Ralf Kittler, Esra A. Akbay, Bruce Posner, Yuzhuo Wang, Stephen Lam, Steven A. Kliewer, David J. Mangelsdorf, John D. Minna

**Affiliations:** ^1^ Department of Internal Medicine, Hamon Center for Therapeutic Oncology Research, University of Texas Southwestern Medical Center, Dallas, TX, United States; ^2^ Department of Pharmacology, University of Texas Southwestern Medical Center, Dallas, TX, United States; ^3^ Department of Biochemistry, University of Texas Southwestern Medical Center, Dallas, TX, United States; ^4^ Eugene McDermott Center for Human Growth and Development, University of Texas Southwestern Medical Center, Dallas, TX, United States; ^5^ Children’s Medical Center Research Institute at University of Texas (UT) Southwestern Medical Center, Dallas, TX, United States; ^6^ Department of Cell Biology, University of Texas Southwestern Medical Center, Dallas, TX, United States; ^7^ Department of Biochemistry and Molecular Genetics, University of Illinois at Chicago, Chicago, IL, United States; ^8^ Department of Urology, Department of Cellular and Molecular Physiology, Yale School of Medicine, New Haven, CT, United States; ^9^ Department of Translational Molecular Pathology, The University of Texas MD Anderson Cancer Center, Houston, TX, United States; ^10^ Department of Thoracic/Head and Neck Medical Oncology, University of Texas, MD Anderson Cancer Center, Houston, TX, United States; ^11^ Simmons Cancer Center, University of Texas Southwestern Medical Center, Dallas, TX, United States; ^12^ Department of Pathology, University of Texas Southwestern Medical Center, Dallas, TX, United States; ^13^ British Columbia Cancer Center, Vancouver, BC, Canada; ^14^ Department of Molecular Biology, University of Texas Southwestern Medical Center, Dallas, TX, United States; ^15^ Department of Pharmacology, Howard Hughes Medical Institute, University of Texas Southwestern Medical Center, Dallas, TX, United States; ^16^ Department of Internal Medicine, University of Texas Southwestern Medical Center, Dallas, TX, United States

**Keywords:** LKB1, glucocorticoid, nuclear receptor, targeted therapy, lung cancer

## Abstract

The glucocorticoid receptor (GR) is an important anti-cancer target in lymphoid cancers but has been understudied in solid tumors like lung cancer, although glucocorticoids are often given with chemotherapy regimens to mitigate side effects. Here, we identify a dexamethasone-GR mediated anti-cancer response in a subset of aggressive non-small cell lung cancers (NSCLCs) that harbor Serine/Threonine Kinase 11 (STK11/LKB1) mutations. High tumor expression of carbamoyl phosphate synthase 1 (CPS1) was strongly linked to the presence of LKB1 mutations, was the best predictor of NSCLC dexamethasone (DEX) sensitivity (*p* < 10^-16^) but was not mechanistically involved in DEX sensitivity. Subcutaneous, orthotopic and metastatic NSCLC xenografts, biomarker-selected, STK11/LKB1 mutant patient derived xenografts, and genetically engineered mouse models with KRAS/LKB1 mutant lung adenocarcinomas all showed marked *in vivo* anti-tumor responses with the glucocorticoid dexamethasone as a single agent or in combination with cisplatin. Mechanistically, GR activation triggers G1/S cell cycle arrest in LKB1 mutant NSCLCs by inducing the expression of the cyclin-dependent kinase inhibitor, *CDKN1C*/p57(Kip2). All findings were confirmed with functional genomic experiments including CRISPR knockouts and exogenous expression. Importantly, DEX-GR mediated cell cycle arrest did not interfere with NSCLC radiotherapy, or platinum response *in vitro* or with platinum response *in vivo*. While DEX induced LKB1 mutant NSCLCs *in vitro* exhibit markers of cellular senescence and demonstrate impaired migration, *in vivo* DEX treatment of a patient derived xenograft (PDX) STK11/LKB1 mutant model resulted in expression of apoptosis markers. These findings identify a previously unknown GR mediated therapeutic vulnerability in STK11/LKB1 mutant NSCLCs caused by induction of p57(Kip2) expression with both STK11 mutation and high expression of CPS1 as precision medicine biomarkers of this vulnerability.

## Introduction

Stratification of lung cancer patients based on tumor genomic, transcriptomic or proteomic characterizations can inform clinical decisions. Although there have been successes with targeted agents in small, well-defined cohorts of lung cancer patients, some of the most common oncogenic driver and tumor suppressor mutations have not been therapeutically addressed. Liver Kinase B1 (LKB1) protein is encoded by the Serine/Threonine Kinase 11 (*STK11)* gene and is the second most commonly mutated tumor suppressor in non-small cell lung cancer (NSCLC) after *TP53* (1–3). Estimates suggest that LKB1/STK11 function is lost in approximately 30% of NSCLC tumors, although this is likely an underestimate of the frequency of loss of LKB1. The LKB1 protein functions as a serine-threonine kinase involved in numerous cellular processes including energy homeostasis, metabolism and cellular polarity exerting its regulatory influence *via* phosphorylation of downstream kinases. Loss of LKB1 confers many potential advantages to lung cancers by removing a central metabolic regulator and energy sensor from the AMPK pathway (4). Mutations in LKB1 commonly co-occur with activating KRAS mutations (often referred to as “KL tumors”) comprising 7-10% of lung adenocarcinoma patients (5–8). The high frequency of LKB1 loss, its significant association with KRAS mutations and resistance to immune checkpoint blockade immunotherapy as well as chemotherapy, targeted therapy has stimulated efforts to identify LKB1-specific vulnerabilities (6). The role LKB1 plays in energy-sensing pathways makes it an attractive target and initial efforts targeting KL tumors focused on biguanide drugs like metformin (9). Recent studies have identified novel metabolic targets, including carbamoyl phosphate synthase 1 (CPS1) in KL tumors and glutaminase (GLS) in KL tumors that also have Kelch Like ECH Associated Protein 1 (KEAP1) mutations (10, 11).

Although the high rate of co-occurrence with KRAS has brought attention to LKB1 mutations, it is important to note that it is more often than not found without other well characterized driver mutations (1). In the last few years, there has been significant focus on identifying vulnerabilities in pathways downstream of LKB1 and a number of targets, including mTOR, MEK, PI3K and ERK have been studied (12–15). The identification of vulnerabilities associated with STK11 mutations with or without other driver mutations is important. New NSCLC patients generally undergo CLIA certified tumor mutation analysis to help guide therapy and some diagnostic oncology panels such as Foundation One already include STK11 mutations (www.foundationmedicineasia.com/content/dam/rfm/apac_v2-en/FOne_Current_Gene_List.pdf). As commercial panels continue to develop, identification of STK11 alterations will become more common leading to the identification of thousands of lung and cervical cancer patients who could potentially benefit from STK11 targeted therapy.

The emergence of immune checkpoint inhibitors has encouraged thorough characterization of the immune microenvironment for various lung cancer oncogenotypes in search of predictive biomarkers. While KRAS-TP53 mutant tumors (KP) are known to engage the PD-1/PD-L1 checkpoint, KL tumors in GEMMs secrete pro-inflammatory, immune suppressive cytokines/chemokines and are considered “immune-cold” with little to no PD-L1 expression and poor T-cell involvement (5, 16, 17). Recent observations by Skoulidis et al. indicate that KL tumors are associated with non-response to immune checkpoint (PD-1/PD-L1) inhibitors (6). In addition, Kitajima and colleagues reported that LKB1 loss resulted in epigenetic silencing of stimulator of interferon genes (STING) expression and insensitivity to cytoplasmic double stranded DNA indicating a deficit in innate immunity in these tumors as well (18). Thus, although LKB1 loss and its effects on metabolic dysregulation in cancer have been thoroughly investigated, it is increasingly clear that LKB1 loss also has significant consequences related to immune dysfunction and the tumor microenvironment. Thus, it was of interest that our recent discovery demonstrated that STK11 mutant NSCLCs treated with AXL inhibitors (targeting AXL in the tumor microenvironment dendritic cells) can now respond to immune checkpoint blockade therapy (19).

The CDKN1C gene is an imprinted locus encoding the p57(Kip2) protein, a member of the CIP-KIP family of cell cycle inhibitors which also includes the more well-known p27(Kip1) and p21(Cip1) (20). The p57(Kip2) protein binds tightly to and inhibits cyclin dependent-kinase (CDK) complexes thereby preventing cells from passing the restriction point (R-point) and committing to cell-cycle progression. Transcription from the CDKN1C locus relies on complex cis- and trans-acting control mechanisms involving imprinting centers, non-coding RNAs and epigenetic modifications (21). The role of CDKN1C as a cell cycle inhibitor and its observed downregulation in many cancers has led to its designation as a tumor suppressor gene (TSG). However, somatic mutations in CDKN1C are rare, with expression loss nearly always related to epigenetic inactivation making it an ideal candidate for targeted re-expression. Several drugs targeting the cell cycle, including FDA-approved palbociclib, are emerging in the cancer clinic so the potential to activate p57(Kip2) should be of great interest.

Nuclear receptors (NRs) have always been at the vanguard of clinical cancer diagnostics and therapy. Previous work from our group has shown that NRs have both prognostic and therapeutic potential in lung cancer, but there are currently no anti-cancer NR ligands in the lung cancer clinic and their prospective utility remains underexplored (22). Here, we report the discovery that GR agonists are potential anti-cancer agents against a subtype of NSCLC identified by loss of LKB1 function and high expression of CPS1. Using the potent GR agonist, dexamethasone (DEX), we discovered GR agonists cause G1/S cell-cycle arrest *via* up-regulation of CDKN1C in responder NSCLC lines and that growth inhibition occurred in lung adenocarcinoma and squamous tumor lines with and without other oncogenic mutations such as KRAS. DEX inhibited *in vivo* growth in subcutaneous xenografts as a single agent with similar efficacy as cisplatin including a biomarker selected patient derived xenograft (PDX) model, restricted the growth of multiple metastatic lesions in an orthotopic xenograft model, and showed significant anti-cancer activity in KL genetically engineered mouse models (GEMMs) of lung cancer.

## Results

### Nuclear receptor ligand screen reveals glucocorticoids cause growth arrest in a subset of NSCLC cell lines

**Figure 1:**
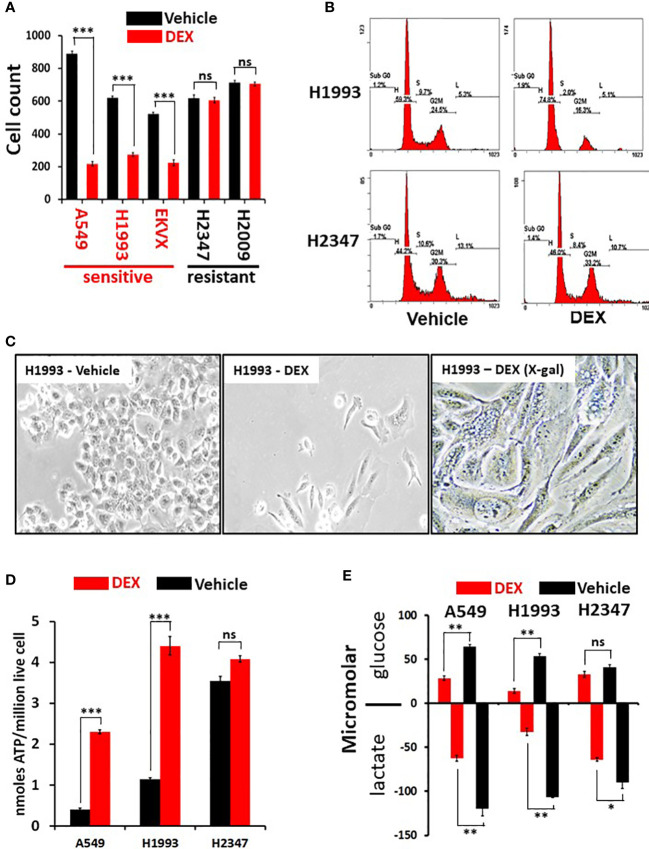
Dexamethasone (DEX) causes growth inhibition and significant metabolic alterations in some lung cancer cell lines. **(A)** Cell counting assays show responder cell lines are significantly growth inhibited by dexamethasone (100nM) over a 5-day assay. Data shown from three independent assays with three replicates for each assay with standard deviation on each bar in graph. Triple asterisk (***) represent p-values < 1x10^-5^ calculated by Student’s t-test. **(B)** After 48 hours of 100nM DEX exposure, DEX sensitive cell line (NCI-H1993) undergoes cell-cycle arrest at the G1/S phase transition as demonstrated by FACS analysis. DEX resistant cell line (NCI-H2347) shows no abnormality in cell cycle distribution in response to drug. FACS assays were repeated three times. **(C)** DEX sensitive cell lines (NCI-H1993) show significantly altered morphology 48 hr. after 100nM DEX exposure with a significant proportion of senescent cells (H1993-DEX (X-gal)). **(D)** DEX sensitive cell lines show significant ATP accumulation in response to DEX exposure while DEX resistant NCI-H2347 does not. Assays done in triplicate with three technical replicates for each assay. Triple asterisk (***) represent p-values < 1x10^-5^ calculated by Student’s t-test. **(E)** DEX sensitive cell lines (NCI-H1993, A549) take up less glucose and excrete less lactate in response to DEX exposure. Resistant cell line NCI-H2347 exhibits no significant reduction in glucose uptake of lactate excretion. Assay done in triplicate. Double asterisk represents p-value < 1x10^-4^ and single asterisk represents p-value < 1x10^-3^ calculated by Student’s t-test. * P < 0.05, ** P < 0.01, ns, not significant.

To comprehensively examine therapeutic leads in lung cancer, 94 NSCLC patient derived lung cancer cell lines were screened in a high-throughput assay to assess for growth response against a drug library consisting of 110 nuclear receptor (NR) ligands (**Table S1**). The NR superfamily consists of 48 members, of which only some are expressed in lung tumors and only a portion of those is known to be ligand activated (in yellow, **Figure S1**). Surprisingly, we observed a significant growth inhibitory effect with GR agonists while the other NR ligands had little effect on NSCLC *in vitro* growth (**Table S1**). In addition, analysis of DepMap data (https://depmap.org/portal/) show that functional genomic knockdown of NR expression in lung cancer lines *in vitro*, including those with STK11 mutations, showed little growth inhibitory or growth stimulatory effects which includes knockdown/knockout of GR (NR3C1 gene). To further understand the growth inhibitory responses in NSCLCs by GR targeted drugs we focused our investigation on the clinically important drug dexamethasone (DEX). We validated our screen data that identified DEX “sensitive” (growth inhibited by DEX) vs. DEX “non-responder” (not growth inhibited by DEX treatment) using five NSCLC lines and demonstrate cell growth was significantly reduced in the three DEX sensitive cell lines (A549, NCI-H1993 and EKVX) while remaining unchanged in the two non-responder cell lines (NCI-H2009 and NCI-H2347, **Figure 1A**). Importantly, growth inhibition was seen using clinically achievable concentrations of DEX (e.g. at 100 nM). DEX mediated growth inhibition was also observed in 3-D spheroid cultures demonstrating ligand bound GR effects are not dependent on 2-D culture conditions (**Figures S2A, B**). We verified that DEX acted through GR using 3 different CRISPR knockouts of GR in DEX-sensitive cell line NCI-H1993 (**Figure S2C**). These three GR knockout clones rescued the growth inhibition response to DEX in cell counting assays (**Figure S2D**) and colony formation assays (**Figure S2E**). Interestingly, loss of GR expression resulted in no observable effects on long term 2-D growth of NSCLC lines like NCI-H1993 which agrees with DepMap data which did not show common dropout of GR (NR3C1) in NSCLC lines. Loss of GR expression in our knockout model (NCI-H1993 - GR2-7) did not alter the platinum or radiation response in comparison to its parental cell line *in vitro* (**Figures S2F–H**). FACS and morphology analyses in DEX-sensitive NSCLC lines revealed that cells were growth arrested at the G1/S transition and had undergone substantial changes in cell shape suggesting a senescent phenotype which was confirmed by beta-galactosidase staining (**Figures 1B, C**, **S3A**). Gene expression analyses further support the conclusion that DEX-induced morphology changes in responder NSCLC lines were not the result of EMT pathway activation. Collagen invasion assays demonstrated that DEX treated cells were not only growth inhibited but less migratory than untreated cells (**Figure S3B**). Furthermore, DEX treated, growth arrested cell lines showed significant accumulation of ATP and reduced rates of glucose utilization with a concomitant reduction in lactate excretion (**Figures 1D, E**) also indicative of a senescent phenotype.

### GR ligand response predicted by high expression of CPS1 in LKB1-mutant lung cancers

**Figure 2:**
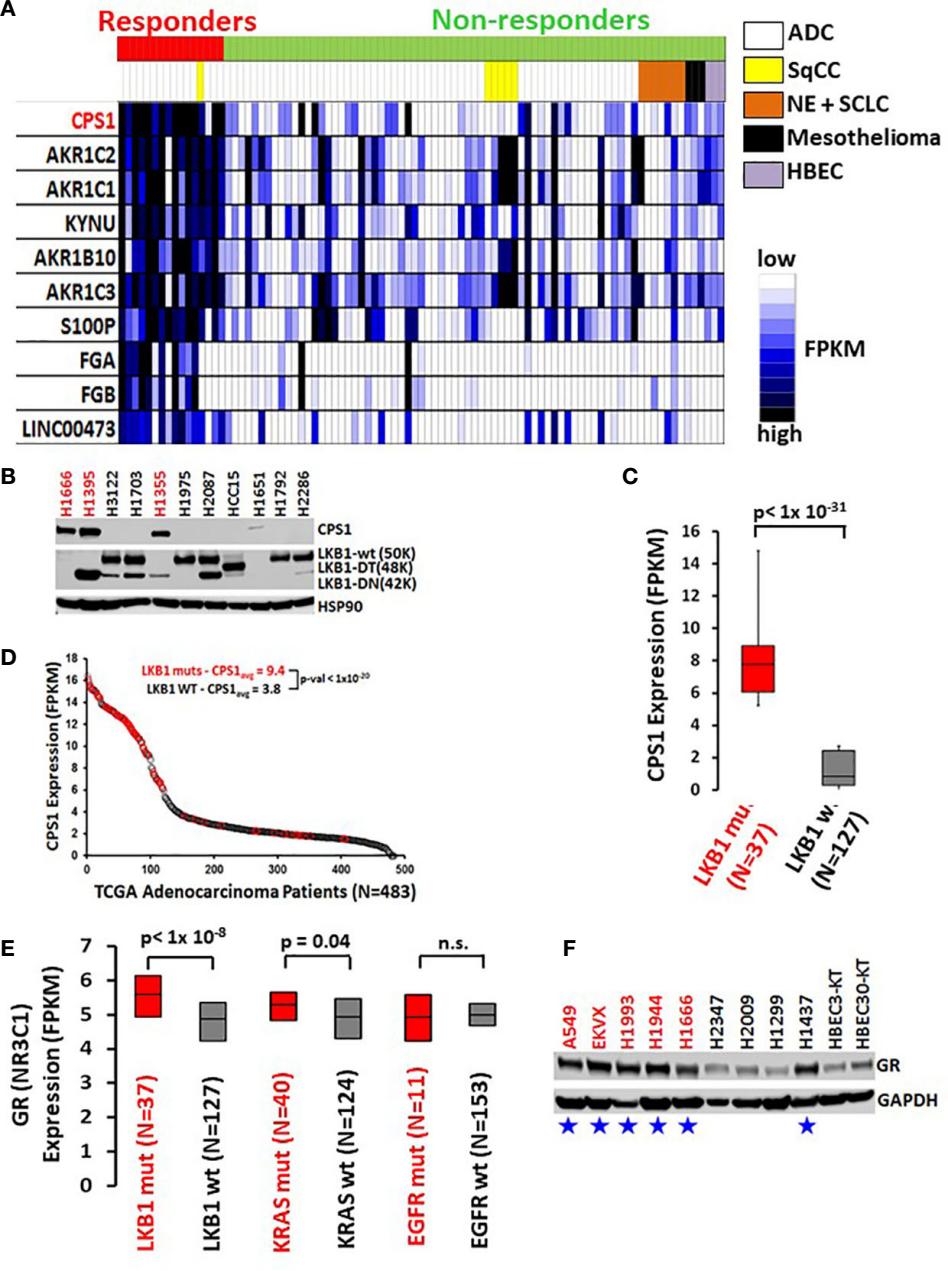
High CPS1 expression is the biomarker for DEX sensitivity and LKB1 mutation in lung cancer cell lines. **(A)** RNA expression heatmap of cell lines from NR ligand screen. CPS1 (red) was the most highly expressed gene in the DEX sensitive cell lines when compared to DEX resistant cell lines (p < 9.0 x 10^-16^). **(B)** Exemplar western blot demonstrating the inverse correlation between the presence of LKB1 WT protein (50KDa) and the expression of CPS1 in lung cancer cell line panel. **(C)** Box and whisker plot of RNAseq analysis demonstrating strong association (p< 1x 10^-31^) of high CPS1 expression with LKB1 mutation in large panel of NSCLC cell lines (N=164) **(D)** Analysis of TCGA transcriptome and mutation data demonstrate LKB1 loss is strongly associated with high CPS1 expression. **(E)** Box and whisker plot of RNAseq data from 164 NSCLC cell lines showing strong correlation between LKB1 loss and increased GR expression (p < 1x10^-8^) and no correlation between GR expression and either KRAS or EGFR mutation. **(F)** Exemplar western blot confirms GR protein is more highly expressed in LKB1 mutants than wild type. Blue stars mark LKB1 mutant cell lines and red font indicates DEX sensitive cell lines.

Selective response to GR agonists in our initial screen led us to examine potential biomarkers predicting sensitivity. A comparison of whole transcriptome RNAseq expression indicated that carbamoyl phosphate synthase 1 (CPS1) was expressed almost 30-fold higher in DEX responders versus non-responders and was by far the best predictor of DEX sensitivity (*p* < 10^-16^, **Figure 2A**). Previous work from our group and collaborators established a strong link between elevated expression of CPS1 and LKB1 mutation and we confirmed this by immunoblot for CPS1 in a selected cell line panel as well as RNAseq data from our own cell line archive and the TCGA dataset (**Figures 2B–D**) (10). Remarkably, CPS1 does not appear to contribute mechanistically to DEX sensitivity as CRISPR knockout of CPS1 did not affect GR mediated growth inhibition response in LKB1 mutant cell line A549 (**Figures S3C, D**). Finally, it was also of interest that, 4 of the top 10 genes associated with DEX response (**Figure 2A**) in our screen are aldo-keto reductases (AKR1C1, AKR1C2, AKR1B10 and AKR1C3) which are involved in steroid metabolism.

**Figure 3:**
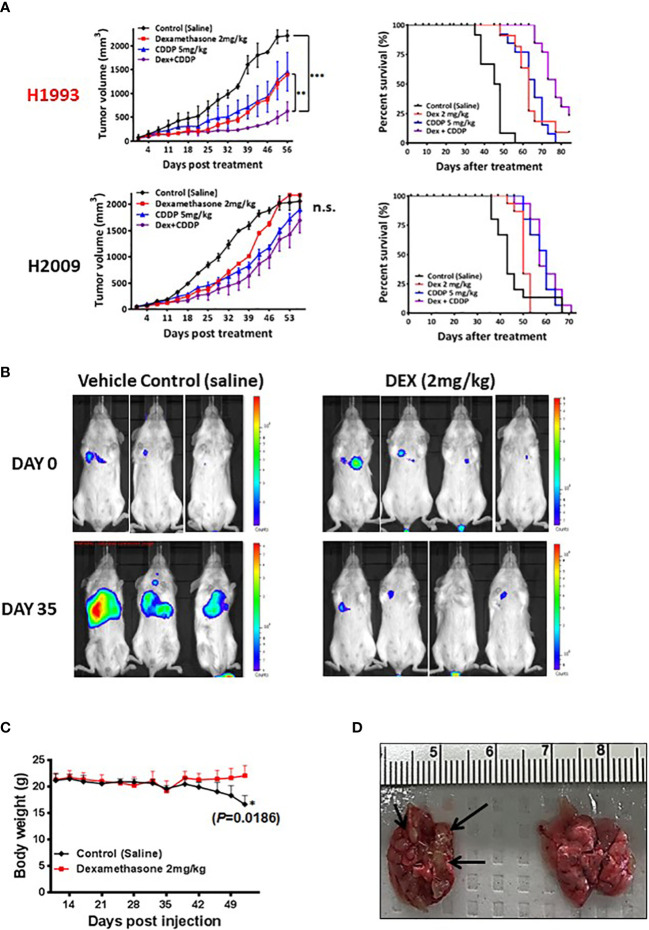
*In vivo* xenograft and orthotopic model studies show DEX mediated inhibition of tumor growth. **(A)** NSCLC cell line xenograft studies using DEX sensitive cell line (NCI-H1993 in red) and resistant cell line (NCI-H2009). Once tumors for each cell line reach an average size of 150 mm3, they are randomized and split into four treatment groups (1); vehicle control (2), DEX only (3), cisplatin only and (4) DEX + cisplatin. Treatments are stopped after four weeks and tumors allowed to progress until they reach ~2000 mm3 at which point mice are sacrificed. DEX and platinum as single agents show similar anti-tumor responses and survival benefit (red, blue line respectively). The combination DEX + cisplatin treated groups performed best in sensitive NCI-H1993 (purple line). No significant response was observed in DEX resistant NCI-H2009. **(B)** Mouse orthotopic model created by tail vein injection of luciferase labeled A549 cells into 20 mice. After three weeks, 7 mice showed similarly sized tumors as judged by BLI. Mice were randomized and split into two groups (1); vehicle control and (2) DEX treated. The first BLI images shown were taken before first day of DEX or vehicle treatment. The second set of images was taken 35 days after the start of treatment. **(C)** Body mass of DEX treated (red line) and vehicle control (black line) during progression of experiment. **(D)** Example of lungs removed after sacrifice for DEX treated (right) and control (left). Black arrows point to visibly significant tumor nodules in vehicle group.

To further validate the predictive value of CPS1 as a biomarker, we characterized CPS1 status of 50 NSCLC cell lines by immunoblot and their DEX-responsiveness in a colony formation assay (**Table 1**). Results show near perfect correlation between CPS1 expression, LKB1 mutant status and DEX growth response. Growth inhibition by DEX was seen in almost all NSCLC subtypes that expressed CPS1, including adenocarcinomas (ADC), squamous cell carcinomas (SqCC). Since NSCLC patients frequently receive platin-doublet chemotherapy (such as platin + taxane) or radiation therapy, it was important to see if treatment with DEX influenced these responses. From **Table 1**, we highlight one NSCLC cell line, NCI-H1355(T16), developed from previous work from our group which was selected to be highly resistant to platinum-taxane doublet chemotherapy both *in vitro* and *in vivo* (23). The NCI-H1355(T16) cell line carries both KRAS (G13C) mutation and an LKB1 mutation and exhibited the same degree of DEX mediated inhibition as the NCI-H1355 platinum-taxane sensitive parental line from which it was derived. Therefore, GR agonist responses are not likely to be altered by other chemoresistance mechanisms which establish resistance to standard platin-taxane doublet chemotherapy. We also investigated potential effects DEX mediated growth inhibition may have on radiosensitivity and found no change in radiotherapy response in either responder (NCI-H1993) or non-responder (NCI-H2009) cell lines (**Figure S4**)

**Table 1:** Summary of results from colony formation assays with 50 NSCLC cell lines after DEX treatment examining growth inhibition, morphology change, mutation status of LKB1 (NGS and western blot) and presence of CPS1 (western blot).

Cell Line (N=50)	NSCLC Subtype	Growth Inhibition	LKB1 Mut	WT LKB1	CPS1
**A549**	**ADC**	**Yes**	**Mut**	**No**	**Yes**
**EKVX**	**ADC**	**Yes**	**WT**	**Yes**	**Yes**
**H1355**	**ADC**	**Yes**	**Mut**	**No**	**Yes**
**H1355 (T16)**	**ADC**	**Yes**	**Mut**	**No**	**Yes**
**H1395**	**ADC**	**Yes**	**Mut**	**No**	**Yes**
**H1437**	**ADC**	**Yes**	**Mut**	**No**	**Yes**
**H1573**	**ADC**	**Yes**	**Mut**	**Yes**	**Yes**
**H1944**	**ADC**	**Yes**	**Mut**	**No**	**Yes**
**H2023**	**ADC**	**Yes**	**Mut**	**No**	**Yes**
**H2030**	**ADC**	**Yes**	**Mut**	**No**	**Yes**
**H2073**	**ADC**	**Yes**	**Mut**	**No**	**Yes**
**H2126**	**ADC**	**Yes**	**Mut**	**No**	**Yes**
**H23**	**ADC**	**Yes**	**Mut**	**No**	**Yes**
**HCC2302**	**ADC**	**Yes**	**Mut**	**No**	**Yes**
**HCC515**	**ADC**	**Yes**	**Mut**	**No**	**Yes**
**H2122**	**NSCLC**	**Yes**	**Mut**	**No**	**Yes**
**H2172**	**NSCLC**	**Yes**	**Mut**	**No**	**Yes**
**HCC1359**	**NSCLC**	**Yes**	**WT**	**Yes**	**No**
**H1666**	**SqCC**	**Yes**	**Mut**	**No**	**Yes**
**HCC1313**	**SqCC**	**Yes**	**Mut**	**No**	**Yes**
**H1651**	**ADC**	**No**	**Mut**	**No**	**Yes**
**H1373**	**ADC**	**No**	**WT**	**Yes**	**No**
**H1693**	**ADC**	**No**	**WT**	**Yes**	**No**
**H1703**	**ADC**	**No**	**WT**	**Yes**	**No**
**H1975**	**ADC**	**No**	**WT**	**Yes**	**No**
**H2009**	**ADC**	**No**	**WT**	**Yes**	**No**
**H2087**	**ADC**	**No**	**WT**	**Yes**	**No**
**H2286**	**ADC**	**No**	**Mut**	**Yes**	**No**
**H3122**	**ADC**	**No**	**WT**	**Yes**	**No**
**H358**	**ADC**	**No**	**WT**	**Yes**	**No**
**H522**	**ADC**	**No**	**WT**	**Yes**	**No**
**HCC1171**	**ADC**	**No**	**WT**	**Yes**	**No**
**HCC15**	**ADC**	**No**	**Mut**	**Yes**	**No**
**HCC2108**	**ADC**	**No**	**Mut**	**Yes**	**No**
**HCC4058**	**ADC**	**No**	**WT**	**Yes**	**No**
**H460**	**NSCLC**	**No**	**Mut**	**No**	**Yes**
**A427**	**NSCLC**	**No**	**WT**	**No**	**No**
**DFCI-024**	**NSCLC**	**No**	**WT**	**Yes**	**No**
**H1792**	**NSCLC**	**No**	**WT**	**Yes**	**No**
**H647**	**NSCLC**	**No**	**Mut**	**Yes**	**No**
**H661**	**NSCLC**	**No**	**WT**	**Yes**	**No**
**HCC366**	**NSCLC**	**No**	**WT**	**No**	**No**
**HCC44**	**NSCLC**	**No**	**Mut**	**No**	**No**
**H1299**	**NSCLC**	**No**	**WT**	**Yes**	**No**
**H2110**	**NSCLC**	**No**	**WT**	**Yes**	**Yes**
**H1755**	**NSCLC-NE**	**No**	**Mut**	**No**	**Yes**
**H1155**	**NSCLC-NE**	**No**	**WT**	**Yes**	**No**
**H1650**	**SqCC**	**No**	**WT**	**Yes**	**No**
**H596**	**SqCC**	**No**	**WT**	**Yes**	**No**
**HCC2344**	**SqCC**	**No**	**WT**	**Yes**	**No**

"red" indicates growth inhibited; "yellow" indicates mutant LKB1 ; "green" indicates CPS1 exporession.

The DEX sensitivity of LKB1 mutant NSCLC cells led us to look for an association between GR expression and other familiar lung cancer oncogenotypes. Analysis of numerous NSCLC cell lines, xenograft and patient datasets using RNA expression from multiple platforms led to the identification of a higher GR transcript expression specifically associated with LKB1 abnormalities (**Figure 2E**). Higher GR protein expression in LKB1 mutant cell lines was confirmed by immunoblotting (**Figure 2F**). We did not observe statistically relevant associations between GR expression and other common lung cancer driver mutations, including KRAS and EGFR.

### DEX inhibits growth of subcutaneous and orthotopic/metastatic xenograft tumors

To assess *in vivo* growth inhibition mediated by either DEX alone or DEX combined with common NSCLC chemotherapy regimens, we generated cell line xenografts (CLX) models using a responder cell line (NCI-H1993) and a non-responder model (NCI-H2009) in mice. Cohorts were divided into four treatment groups: control (PBS); DEX alone (2 mg/kg 4X per week for four weeks); cisplatin (CDDP) alone (once per week over two weeks); and the DEX + CDDP combination. Tumor growth and survival curves for the sensitive NCI-H1993 xenograft show that DEX, as a single agent, was as effective as cisplatin therapy (**Figure 3A**). Surprisingly, the combination of DEX and cisplatin resulted in a statistically superior survival response over each drug alone (**Figure 3A**). The resulting additivity of DEX + CDDP is relevant, as it demonstrates that DEX-mediated cell-cycle arrest does not alter the efficacy of cisplatin therapy. DEX had no effect on CLX tumor growth in resistant cell line NCI-H2009 and did not inhibit efficacy of the platinum study arm. The CLX experiments were repeated three times with identical outcomes.

We next investigated DEX efficacy in an orthotopic/metastatic mouse model using a DEX sensitive cell line harboring luciferase (A549-luc). The cell line was expanded, harvested and injected *via* tail vein into mice. After two weeks, the cell line successfully colonized the lung and tumor growth was monitored using bioluminescent intensity (**Figure 3B**). We chose a cohort of mice (n = 7) with tumors of appropriate and consistent size, randomized them and then treated with vehicle control or DEX. Bioluminescence image analysis revealed lung tumor growth was markedly inhibited in DEX-treated versus vehicle treated control mice (**Figure 3B**), which was confirmed by H&E staining (**Figure S5**). Body weight measurements showed control, but not DEX-treated, mice had significant weight loss associated with increased lung tumor burden (**Figures 3C, D**). Vehicle treated mice exhibited signs of severe discomfort with ruffed fur and labored breathing while DEX treated mice appeared normal.

### DEX inhibits tumor growth in biomarker-selected patient-derived xenograft and genetically engineered mouse models

**Figure 4:**
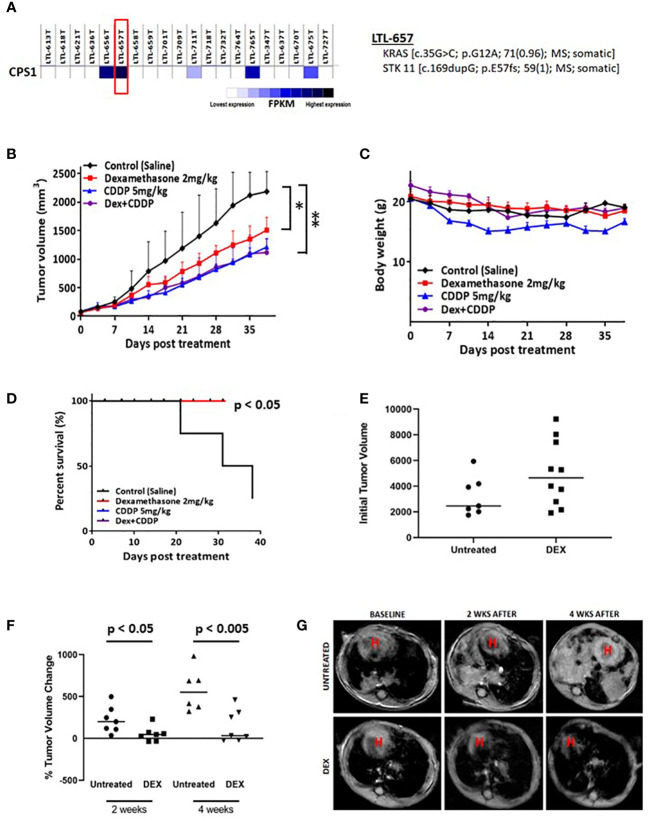
Biomarker driven choice of patient derived xenograft (PDX) and KL tumor GEMMs show response to DEX. **(A)** RNAseq data confirming high expression of CPS1. DNA seq data confirms and characterizes LKB1 mutation. **(B)** High CPS1 expressing PDX tumors were grown to approximately 250mm3 before DEX (red), platinum alone (blue), combination (purple) or vehicle (black) treatment started. Tumor growth curve for PDX tumors treated for five weeks with DEX are shown. **(C)** Average body weight measurement for members of each cohort is plotted over the duration of the experiment. **(D)** Survival curves for each treatment group are plotted. **(E)** MRI estimated tumor volumes for mouse KL GEMM at baseline before DEX treatment. **(F)** MRI estimated tumor volumes plotted as percentage change in tumor volume compared to baseline at two weeks and four weeks. **(G)** Representative MRI of one control treated mouse and one DEX treated mouse over the course of the four week treatment. * means p-value < 0.05; ** means p-value < 0.01.

To further establish the clinical relevance of the synthetic lethality of DEX in LKB1-mutant lung cancer, we chose a NSCLC PDX adenocarcinoma (LTL-657) from a bank of NSCLC PDXs based on high expression of CPS1 and somatic mutations of KRAS and LKB1 (**Figure 4A**). The PDX tumors were initially propagated in donor mice, harvested, dissected and equal sized pieces transplanted into a second set of mice (N=20). PDXs were allowed to grow to 250 mm^3^, randomized and divided into four treatment groups (vehicle, DEX alone, CDDP alone and DEX + CDDP). As a single agent, DEX significantly inhibited tumor growth and was nearly as efficacious as cisplatin alone (**Figure 4B**). Although further inhibition of tumor growth was not observed in the DEX + CDDP treatment group, it is noteworthy that as previously observed in the CLX experiment (**Figure 3A**), DEX did not alter the efficacy of cisplatin therapy (**Figure 4B**). Moreover, there was no body weight loss for DEX+CDDP treated mice when compared to untreated, control mice while CDDP alone mice which lost approximately 15% of their body weight on average (**Figure 4C**). Survival curves also showed that mice treated with DEX alone, CDDP alone or in combination survive significantly longer than untreated mice (**Figure 4D**).

We also utilized a previously described mouse GEMM harboring LKB1 deletion and oncogenic KRAS expression (denoted as KL mice) to further demonstrate the therapeutic potential of DEX. KL mice were randomized, and initial tumor volumes were measured by MRI (**Figure 4E**). Mice were treated with DEX (2mg/kg) four days per week for four weeks and tumor growth was monitored by MRI once every two weeks for the duration of the experiment. Measurements after two and four weeks revealed that tumors in DEX-treated mice were static while vehicle-treated mouse tumors grew rapidly (**Figures 4F, G**). Taken together the above studies suggest the therapeutic potential of using glucocorticoids to treat LKB1-mutant lung tumors and provide CPS1 expression as a robust biomarker to predict responsiveness.

### *CDKN1C* mediates glucocorticoid sensitivity in LKB1 mutant lung cancer

**Figure 5:**
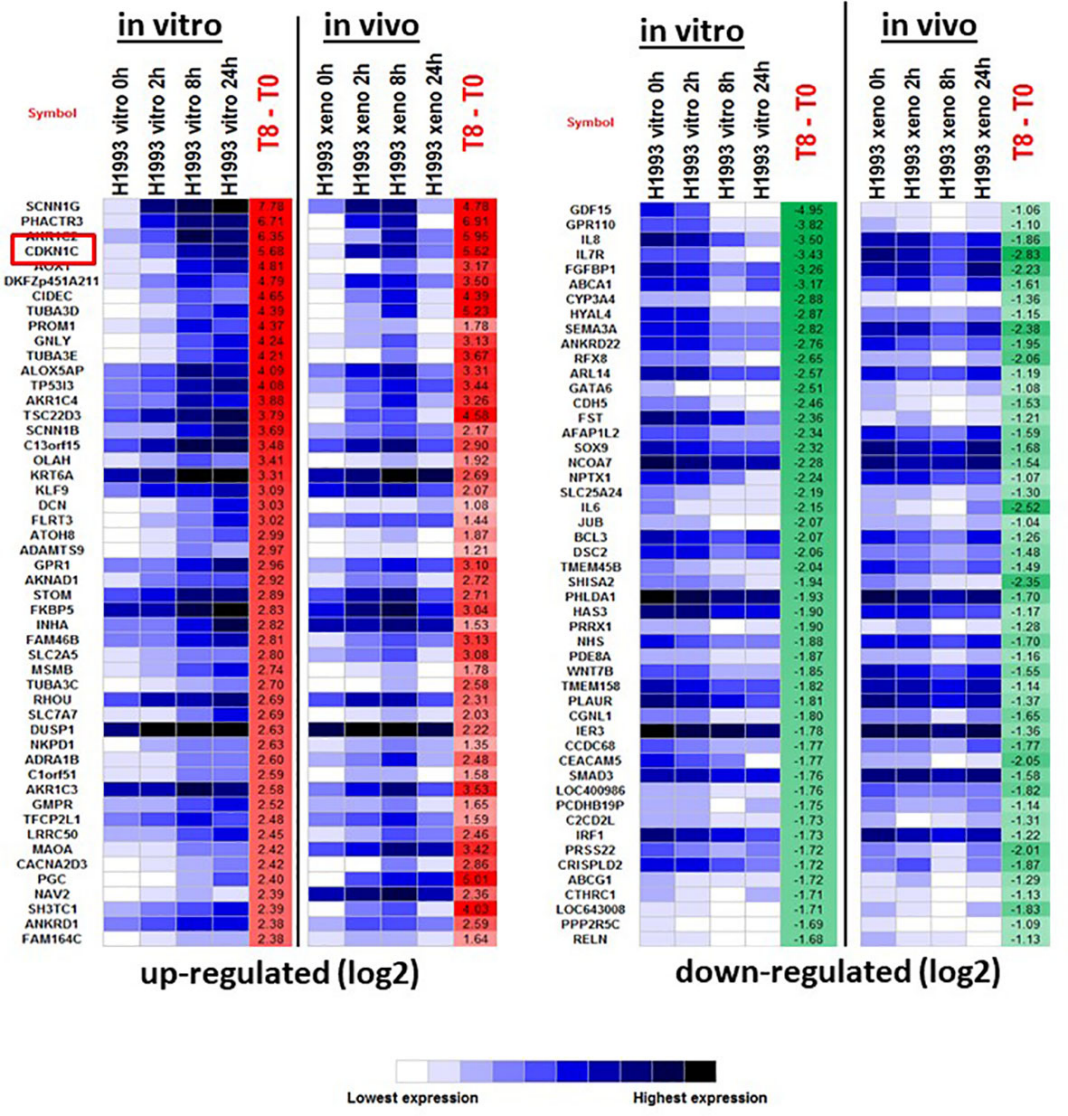
The top 50 up- and down-regulated genes in response to DEX in DEX-sensitive NCI-H1993 *in vitro* and *in vivo* (xenograft). CDKN1C is highlighted.

**Figure 6:**
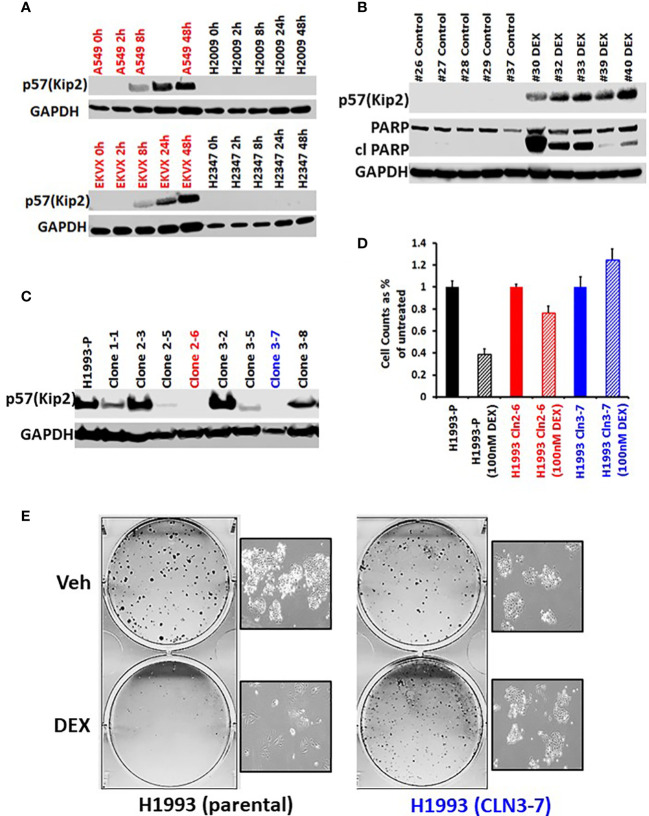
GR mediated up-regulation of p57(Kip2) expression is responsible for the cell cycle arrest in DEX-sensitive cells. **(A)** Western blot showing time course of p57(Kip2) protein appearance after DEX treatment in sensitive (A549, EKVX) and resistant (NCI-H2009, NCI-H2347) cells. **(B)** Western blot of LKB1 mutant PDX tumor exhibiting p57(Kip2) expression and the appearance of cleaved PARP in the DEX treatment group. **(C)** Western blot of NCI-H1993 CRISPR clones and parental (H1993-P) after DEX treatment (100nM, 24 hrs.). NCI-H1993 CRISPr clones 2-6 (red) and 3-7 (blue) show no p57(Kip2) after DEX treatment and were used in subsequent cell proliferation assays. **(D)** Cell counts after DEX treatment with parental NCI-H1993 and CRISPR knockout clones NCI-H1993 cln2-6 and cln3-7. **(E)** Colony formation assay confirming loss of cell cycle arrest after CRISPR knockout of CDKN1C gene in DEX-sensitive NCI-H1993. Inset shows exemplar brightfield images of colonies from the colony formation assay plates shown.

To investigate the mechanism of GR-mediated inhibition of LKB1 mutant tumors, we analyzed the transcriptome of the responder NCI-H1993 cancer cells both *in vitro* and *in vivo*. RNA was extracted from either NCI-H1993 cultured cells or mouse xenograft tumors after treatment with DEX for 2, 8 and 24 hours. Whole transcriptome analysis revealed that the cross section of genes regulated by DEX *in vitro* and *in vivo* was similar (**Figure 5**). Of particular interest, we noted the strong DEX-dependent upregulation of *CDKN1C* expression. *CDKN1C* is a known GR target gene and encodes the cyclin-dependent kinase inhibitor p57(Kip2). Western blot analysis of LKB1 mutant cell lines (NCI-H1993, EKVX) confirmed p57(Kip2) protein expression was induced after exposure to DEX, while LKB1 wild-type cell lines (NCI-H2009, NCI-H2347) showed no p57(Kip2) expression over the course of the experiment (**Figure 6A**). Moreover, p57(Kip2) protein expression was also upregulated in all the PDX tumors treated with DEX (**Figure 6B**). Coincident with p57 expression, PARP cleavage was detected in all DEX treated samples as a biomarker of apoptosis (**Figure 6B**). Chromatin immunoprecipitation (ChIP) assays with GR antibody validate the occupancy of glucocorticoid response element (GRE) in DEX sensitive lung cancer cell lines and is in close proximity (25KB) to the CDKN1C promoter of DEX sensitive cell lines (**Table S2**).

To confirm the role of p57(Kip2) in growth inhibition of sensitive cell lines we used CRISPR to knock out *CDKN1C* in NCI-H1993. Two clones showed complete loss of DEX-dependent p57(Kip2) expression (**Figure 6C**) and were refractory to DEX inhibition of cell growth (**Figure 6D**) and colony formation (**Figure 6E**). Although *CDKN1C* was not induced by DEX in LKB1 wild-type cell lines, we tested whether exogenous expression of *CDKN1C* in NSCLC as well as SCLC cell lines could induce growth inhibition. Doxycycline inducible *CDKN1C* expression vectors and empty control vectors were transfected into DEX-resistant NSCLC (NCI-H1299, NCI-H2009) and SCLC cell lines (NCI-H2081, NCI-H446). Expression of p57(Kip2) was confirmed by immunoblot (**Figures S6A, C**) and analysis of these derivatives demonstrated that p57(Kip2) expression was able to dramatically reduce colony forming ability (**Figures S6B, D**) demonstrating that GR mediated activation of p57 expression is LKB1 dependent, but that cell cycle inhibition is not.

## Discussion

### Vulnerability identification

Beginning by screening 110 NR ligands against a panel of 90 patient derived NSCLC lines, we found a subset that are dramatically growth inhibited both *in vitro* and *in vivo* by multiple GR agonists. Glucocorticoid responsive cell lines undergo cell cycle arrest, exhibit significant non-EMT related morphologic changes, are significantly less invasive and exhibit significant metabolic changes (intracellular accumulation of ATP and reduction of both glucose uptake and lactate release). We showed this growth and metastases inhibitor phenotype is restricted to LKB1/STK11 mutated NSCLCs, is mediated specifically by the GR receptor, and occurs mechanistically by GR agonists upregulating expression of CDKN1C (p57). How can these findings lead to new therapeutic targeting of vulnerabilities for lung cancer treatment? Current estimates suggest nearly 30% of NSCLC tumors have lost LKB1 making it one of the most mutated genes in lung cancer patients and the subject of intense investigation in recent years. LKB1 loss commonly co-occurs with therapeutically intractable KRAS mutations in lung cancer (KL tumors) and defines an oncogenotype with particularly aggressive features and poor outcomes. In general, targeting tumor suppressors has been a difficult challenge but LKB1 is an attractive target because of its role as master regulator of the AMPK as well as other metabolic pathways. Some reports, including from our collaborators, demonstrate the possibility of targeting metabolic vulnerabilities in KL tumors (1, 4, 14, 15, 24, 25). Moreover, enthusiasm surrounding immune therapy in lung cancer has been tempered by the observation that LKB1 mutant tumors are “immune cold” exhibiting no objective response to immune checkpoint inhibitors and the ability to escape immune surveillance by silencing the STING pathway (6, 18). Given our recent finding of targeting AXL in host dendritic cells, it will be important to test the combination of AXL inhibition with DEX treatment in these models, particularly given the known immune suppressive effects of glucocorticoids (19). In fact, if combining AXL inhibition and dexamethasone enhances anti-tumor immune responses this would be an unexpected result.

### Precision medicine biomarkers

Besides documenting STK11 mutations, transcriptome comparisons of NSCLC DEX responders versus non-responders revealed that high expression of CPS1 is an accurate predictor of GR response. Previous work from our group and collaborators demonstrated CPS1 expression is strongly associated with LKB1 mutation (10). CPS1 is amenable to IHC staining and therefore a potential surrogate biomarker for loss of LKB1 function in NSCLCs. Despite being a strong biomarker of GR agonist vulnerability, CRISPR knockout of CPS1 demonstrated that the protein played no role in GR mediated cell cycle arrest. We were very interested to find that DEX growth inhibition associated with LKB1 loss of function is not NSCLC histologic subtype specific and occurred in both adeno- and squamous carcinoma cell lines harboring high CPS1 expression. Although CPS1 expression positively predicted almost all DEX response phenotypes, there were exceptions (HCC4018 [c.1852G>T; p.A618S; missense; somatic] and HCC15 [c.32G>T; p.R11I; missense; somatic]) where co-occurring inactivating mutations in GR may have blunted DEX response (26). Interestingly, we did not observe growth inhibitory activity with DEX in NSCLC-neuroendocrine (NSCLC-NE) lines, and SCLC lines where GR has been postulated to be a tumor suppressor (27).

### Mechanism defined facilitates future therapy development

Preclinical models, including cell line xenografts, orthotopic and metastatic models demonstrate the ability of DEX to inhibit tumor growth *in vitro* and *in vivo*. Analysis of the transcriptional output of ligand activated GR from both *in vitro* and *in vivo* experiments led to the identification of the cell cycle inhibitor p57(Kip2) (encoded by the CDKN1C gene) as the potential mediator of DEX mediated growth inhibition. P57(Kip2) is a member of the Cip/Kip family of cell cycle inhibitors and has been assigned many functional roles, including proliferation, apoptosis, invasion, metastasis, differentiation and angiogenesis. The role of p57(Kip2) as a gatekeeper preventing commitment past the G1/S transition makes it a very attractive option, especially with the recent focus on CDK4/6 inhibitors like the FDA approved cancer drug palbociclib. We confirmed the key and central role of CDKN1C in this vulnerability through CRISPR knockout of CDKN1C which reversed the cell cycle arrest of DEX sensitive cancer cells while DOX-inducible constructs overexpressing CDKN1C in resistant NSCLC lines produced a strong growth inhibition phenotype. Although the use of glucocorticoids as a long term maintenance therapy for cancer patients is less than ideal, a therapy capable of inducing CDKN1C expression is an attractive strategy not only because of strong growth inhibition but also because somatic inactivating mutations of CDKN1C are rare.

### Efficacy of vulnerability in preclinical models

We examined the clinical relevance of this discovery using two different *in vivo* models. We used a NSCLC adenocarcinoma PDX chosen specifically for its high expression of the CPS1 biomarker and demonstrated (1); DEX as a single agent can inhibit PDX growth (2), DEX elicits upregulation of CDKN1C (3), DEX results in apoptotic response with appearance of cleaved PARP and (4) DEX does not interfere with platinum toxicity and in fact may be additive as seen in cell line xenograft experiments. This finding in our report stands in contrast to published reports suggesting that dexamethasone interferes with the efficacy of clinical chemotherapy regimens in some preclinical models (28–33). We cannot speculate as to the mechanisms behind reduced chemotherapy responses observed in breast and ovarian cancer models as there have not been reports of LKB1 loss in significant percentages of those cancer populations. Moreover, the Conzen group at UT Southwestern has shown evidence that some triple negative breast cancers (TNBCs), which are not LKB1 mutant, activate a glucocorticoid mediated, GR driven transcriptional response that is antagonistic to chemotherapy thus potentially leading to poor outcomes (34). Two other papers, one from Herr et al. and very recent work from Prekovic et al. do highlight results that appear to stand in contrast to our report. The report from Herr at al. highlights adverse responses combining dexamethasone and platinum using a lung epidermoid carcinoma (P693) in a nude mouse model and a cervical carcinoma (P5) *in vitro*. Unfortunately, we are unable to assess the LKB1 status of these two models although LKB1 loss is common in both cervical and lung cancers. However, we do note that we did not find any anti-apoptotic transcriptional signatures activated by dexamethasone in our responder cell lines either *in vitro* or *in vivo*. The second paper from Prekovic et al. was the first to identify GR ligand activation of p57 expression in lung cancer but interestingly GR driven p57 expression was first noted in a 1999 paper from Samuelsson et al. in HeLa cells, which were subsequently shown to be LKB1 deficient (35, 36). While we accept Prekovic et al. data that dexamethasone mediated cell cycle arrest during *in vitro* experiments may compromise the ability of some chemotherapy agents to be effective, they do not highlight reduced efficacy for any platinum agents, and we point out the very high level of efficacy of dexamethasone alone in their NCI-H1944 xenograft model (**Figure 2F** of their paper) which is a LKB1 mutant NSCLC. Finally, DEX as a single agent in a KRAS/LKB1 GEMM model resulted in significant overall tumor growth inhibition and complete tumor regression in one animal. Thus, the LKB1/STK11 generated vulnerability can be demonstrated with both human (PDXs) and mouse (GEMMs) preclinical models.

### Targeting the LKB1/STK11 vulnerability epigenetically

LKB1 is a well-known tumor suppressor not only in lung but in several other tissue types, including cervix, ovary and pancreas. The high frequency of LKB1 loss has spurred significant efforts to identify small molecules targeting LKB1 or its affected pathways. Recent work associating NSCLC patients carrying co-occurring LKB1 and KRAS mutations with poor prognosis and non-response to immune modulatory drugs highlights a significant unmet need in the lung cancer clinic. This report conclusively demonstrates that clinically pertinent GR agonists can effectively target LKB1 mutant lung cancers, regardless of KRAS or KEAP1 status. Prekovic et al. identified a GR mediated inhibition of lung cancer cell line growth mediated by glucocorticoid activation of p57 expression and that responsive cell lines were characterized by specific chromatin interactions that were not present in non-responder cell lines. Considering our findings in this context, it becomes clear that LKB1 loss manifests in substantial modification to the chromatin landscape as evidenced by the availability of the normally repressed CDKN1C promoter and the high expression of CPS1 which is not observed in lung cancers without LKB1 mutations. Work from Kottakis et al. in 2016 demonstrated that co-occurring LKB1 and KRAS mutations in pancreatic cancer GEMMs elicit significant epigenetic changes stemming from aberrant activity in the serine metabolic axis (37). The ability of GR to mediate expression of CDKN1C expression in LKB1 mutant tumors suggests that one roadblock to activating expression in non-responder cell lines is the chromatin restrictions preventing productive enhancer - promoter interactions. Could these restrictions be potentially overcome with epigenetic modifying compounds? The significant alterations to the lung cancer epigenome as a result of metabolic changes brought on by LKB1 loss will most certainly be of interest in the search for tumor vulnerabilities.

### Integrating the LKB1/STK11 vulnerability with immunotherapy, chemotherapy and radiation therapy for NSCLC

When considering the clinical utility of cell cycle repression *via* p57 activation, one of the most significant concerns is that established therapeutic regimens that depend on cell cycle progression may become less effective. Although DEX does not appear to interfere in any way with platinum therapy, we did not examine any potential effects when used in combination with standard platin doublets (platin-taxane, platin-pemetrexed, platin-gemcitabine). DEX mediated growth inhibition also does not appear to interfere with radiotherapy response during *in vitro* cell line colony formation assays with either DEX responders or non-responders.

While most NSCLC patients receiving chemotherapy or radiotherapy are also administered dexamethasone to blunt serious side effects, there would be major concerns with regards to dexamethasone use in the lung cancer clinic as a potential “maintenance therapy” in the same vein as tamoxifen use for breast cancer. One obvious concern is potential reduced efficacy for immunotherapy regimens. Although this may not be relevant for immune cold LKB1 mutant lung tumors, DEX is most well known as an anti-inflammatory agent that almost certainly would suppress anti-tumor immune responses. Although there have been reports endeavoring to address this complex issue, our incomplete understanding of the TME, the dynamics that shape the immune landscape and questions concerning tissue specific immune response leave the issue unsettled. In any event, we and otherse have relevant preclinical models including GEMMs and “humanized mouse” models for PDX studies that can test whether there is impairment of immune checkpoint blockade therapy before proceding to clinical translation. Because of all of the typical concerns with well known long term glucocorticoids side effects, we plan to study the effectiveness of transient and/or intermittent DEX therapy with immune checkpoint blockade in these LKB1 mutant NSCLC preclinical models.

In conclusion, we have discovered a new, acquired vulnerability in NSCLC associated with LKB1/STK11 mutations in a subset of NSCLCs that is represented by GR agonist induced CDKN1C expression leading to a host of anti-tumor responses. The expression of CPS1 and the LKB1/STK11 mutations provide a stable, readily identifiable biomarker for vulnerability identification in precision medicine protocols. This vulnerability mechanistically depends on a targeted therapy to reactivate expression of the CDKN1C gene. Although glucocorticoids may not be the long-term clinical answer to the LKB1 problem, we believe we have identified an oncogenotype specific vulnerability that bears further investigation.

## Materials and methods

### Tissue culture and cell lines

Most NSCLC lines used in this study were part of the NCI and HCC (Hamon Cancer Center at UT Southwestern) series of cell lines, with the exception of A549, Calu.1, Calu.6 (American Type Culture Collection; ATCC), DFCI.024, DFCI.032 (Dana Farber Cancer Institute, courtesy of Pasi Jänne), EKVX, Hop62 (NCI-60 panel), PC9 (Johns Hopkins University School of Medicine, courtesy of Bert Vogelstein). Cell lines from these collections were cultured in RPMI 1640 (GIBCO, 2.05mM L-glutamine) supplemented with 5% FBS (GIBCO). Normal bronchial epithelia-derived cell lines (HBECs, Ramirez et al., 2004) were grown in ACL4 (RPMI 1640 supplemented with 0.02 mg/ml insulin, 0.01 mg/ml transferrin, 25 nM sodium selenite, 50 nM hydrocortisone, 10 mM HEPES, 1 ng/ml EGF, 0.01 mM ethanolamine, 0.01 mM O-phosphorylethanolamine, 0.1 nM triiodothyronine, 2 mg/ml BSA, 0.5 mM sodium pyruvate) with 5% FBS. All cell lines were maintained in a humidified environment in the presence of 5% CO2 at 37°C. All cell lines were were DNA fingerprinted (Powerplex 1.2 Kit, Promega) and mycoplasma free (myco kit, Boca Scientific).

### High throughput screen - NHR ligand cytotoxicity assays

The UT Southwestern NHR ligand library consists of 110 chemicals purchased from Sigma. Details of the molecules, including CAS numbers are listed in **Table S1**. Each NSCLC cell line was cultured in T75 flasks in NSCLC culture medium (RPMI/L-glutamine medium (Invitrogen) and 5% FBS (Atlanta Biologicals) for primary screening. For cell viability assays, cell lines were plated at varying densities determined in previous work in 384 well microtiter assay plates (Bio-one; Greiner). After incubating the assay plates overnight under the growth conditions described above, NR ligands were added to each plate at 12 half-log doses ranging from 50 μM to 50 pM for dose-response studies (3 replicates per dose per cell line). In all experiments, we maintained a final DMSO concentration of 0.5%. After an incubation of 96 hr under growth conditions, CellTiter-Glo reagent (Promega) was added to each well (10 μl of a 1:2 dilution in NSCLC culture medium or ACL4) and mixed. Plates were incubated for 10 min at room temperature and luminescence was determined for each well using an EnVision multi-label plate reader. The assays displayed Z’ values greater than 0.6. This high throughput assay is comprehensively outlined in reference 26.

### Analysis of NR ligand HTS data

Data analysis for this type of screen is comprehensively described in McMillan et al. (26).

### Statistical analysis

All p-values reported in **Figures 1**, **2** result from the Student’s t-test. Log-rank tests were applied to survival analyses (Kaplan–Meier curves). All *in vivo* statistical analyses were performed using GraphPad Prism software. Analyses of covariance (ANCOVA) and nonlinear regression models and overall *P* values were simultaneously calculated, comparing intercepts and slopes for different treatments. All results were considered statistically significant for *P* values of less than 0.01.

### Cell counting assays

Cell lines were seeded and grown for 24 hours in 6-well plates (Corning) in RPMI-1640 with 5% FBS and then treated with 100nM dexamethasone (stock 1mM in PBS) for 96 hours. Cells were trypsinized and counted (Beckman Coulter Z2 Particle Counter at >12 microns). Cell counting assays were done in triplicate.

### Colony formation assays

Cells were counted using a Beckman Coulter Z2 Particle Count and Size Analyzer and plated at a density of 400 cells per well of a 6-well plate. Cells were treated the next day with serial dilutions of dexamethasone. Plates were kept in the cell culture incubator until termination of assay. After 2-3 weeks, colonies were stained with crystal violet staining solution (0.5% crystal violet, 3% formaldehyde solution), rinsed in water and imaged.

### Flow cytometry analysis

For DNA content analysis, cells were seeded at density of 1.5 × 105 per well in 6-well plate and after 24 hr in cell culture with 100nM dexamethasone. Cells were collected by trypsinization, resuspended in 1 mL of ice-cold PBS-F (1 x PBS, 2% FBS), followed by drop-wise addition of 10 mL ice-cold 70% ethanol. Following overnight incubation at 4°C, cells were washed twice with PBTA (1x PBS, 1% BSA, 0.1% Tween-20), stained with propidium iodide (Sigma) containing RNase A at 37°C for 30 min. Fluorescence of the PI-stained cells was measured using a FACSCalibur (BD Biosciences) and analyzed with FlowJo software (BD Bioscience). Assays for dexamethasone sensitive (NCI-H1993) and resistant (NCI-H2347) were done in triplicate with representative data shown in **Figure 1B**.

### Nutrient utilization

To measure metabolic rates, one million cells were plated into 6-cm dishes and cultured until 90% confluent. At time 0, the cells were rinsed in PBS, fed with 1.5 mL of RPMI with 10 mM glucose, 2 mM glutamine and dialyzed FBS, and cultured. End-point experiments proceeded for 7 hours, then the medium was collected and analyzed for metabolite abundance. Concentrations of glucose, lactate, glutamine, and glutamate were determined from 0.6-mL aliquots of medium using an automated electrochemical analyzer (BioProfile Basic-4 analyzer; NOVA). Metabolic rates were determined by normalizing absolute changes in metabolite abundances to final protein content. For estimated metabolic rates normalized to the average protein content over the 7 hour culture period, we assumed exponential growth throughout the 7 hour period and derived an average protein content from the Day3/Day1 and Day5/Day1 cell proliferation data.

### Surviving fractions radiation assays

Surviving Fraction Curves NSCLC and HBEC cells were trypsinized and re-suspended as single cell suspensions. Cells were counted (Z2 Particle Counter, Beckman Coulter) and a fixed number of cells was plated for individual doses of radiation across experimental groups, with increasing number of cells for higher levels of radiation. Counted cells were seeded in 60 mm tissue culture dishes in triplicate for each dose of radiation, allowed to attach to the dish for 6–8 hours, then irradiated at various doses using a 137Cs irradiator (Mark 1–68 irradiator, J.L. Shepherd and associates). Irradiated plates were then incubated until colonies formed, where a colony is defined as ~50 cells. The colonies were fixed and stained with 4% formaldehyde (Fischer Scientific, catalog# 50–980-487) in PBS containing 0.05% crystal violet (MilliporeSigma, catalog# C6158), and colonies were counted manually using a light microscope. The surviving cell fraction was calculated as: (Mean colony counts)/[(cells plated) X (plating efficiency)], in which plating efficiency was defined as (Mean colony counts)/(cells plated for unirradiated control). Curve fitting was performed using the Linear Quadratic equation in SigmaPlot (Systat Software Inc.), and SF2 values are from this fitted curve.

### Spheroid assays

Cell lines were trypsinized, counted, and plated into 96-well U-bottom low adherence plates (Nunclon Sphera, Thermo Scientific). Cells were inoculated between 500-4,000 cells per well depending on growth rate. Spheroids were allowed to form over 48 hr, drug was added, and the plates incubated for an additional 96 hr. Luminescence assays were performed using CellTiter-Glo^®^ 3D cell viability assay (Promega) according to the manufacturer’s instructions. The plates were read on a BMG Labtech FLUOstar^®^ Optima. Assays done twice with 6 technical replicates for each assay.

### Beta-galactosidase assay

Cells were plated at low density in a six well plate and allowed to adhere overnight. The cells were then treated with 100nM dexamethasone for 48 hours. Cells were fixed and analyzed using the Cell Signaling Senescence beta-Galactosidase Staining Kit (product number #9860) as directed. X-gal stained cells were imaged using a light microscope (40X).

### MTS assay

For experiments involving dose-response curves for NCI-H1993 and NCI-H1993-GR2-7 with viability readouts, 1000 cells were plated into each well of a 96-well tissue culture treated plate. After 24 hours, cisplatin was added as 8-point serial dilutions. After 4 more days, viability was determined using CellTiter 96 Aqueous One Solution Cell Proliferation Assay (MTS) (Promega, catalog# G3582) on a Molecular Devices SpectraMax 190 microplate reader. Curve fitting and IC50 determination was performed in Excel.

### Immunoblots

Cells were washed twice with ice-cold PBS and then scraped on ice. Cells were lysed with a modified RIPA buffer (50 mM Tris, 150 mM NaCl,.1% SDS, 1% IGEPAL CA-630, 1% sodium deoxycholate, 2 mM MgCl2, pH 8) with 1 unit/μL benzonase (MilliporeSigma, catalog # E1014), protease inhibitors (MilliporeSigma, catalog # P8340) and phosphatase inhibitors (MilliporeSigma, catalog # 4906845001) by rotating lysates at 4°C for 2 hours. Lysates were then cleared by spinning at max speed for 10 minutes, quantified using BCA (ThermoFisher Scientific, catalog # 23225), mixed with 2X Laemmli buffer (BioRad, catalog # 1610737EDU) and boiled for 5 minutes immediately prior to loading. For ATR, ATM, and DNA-PKCS, 20-25 μg of protein was ran on a NuPAGE 3-8% Tris-Acetate gel (ThermoFisher Scientific, catalog # EA0375BOX) using NuPAGE Tris-Acetate SDS Running Buffer (ThermoFisher, catalog # LA0041) at 150V. For all other proteins, 20-25 μg of protein was ran on a 4-20% Mini-PROTEAN TGX gel (BioRad, catalog # 4561096) at 220 V. Samples were transferred using the Trans-Blot Turbo RTA Mini Nitrocellulose transfer kit (Biorad, catalog # 1704270) on the Trans-Blot Turbo Transfer System (Biorad, catalog # 1704150) using manufacturers suggested protocols. For all chemiluminescent blots (**Figures S1F**, **S1H**, **S1J**, **S6A**), blocking/antibody incubation steps were done with 5% milk (Biorad, catalog # 1706404XTU) in.1% in PBST. All other blots were done using near infrared fluorescence, and blocking/antibody incubation steps were done with TBS Odyssey Blocking (LiCor, catalog # 927-50100) with 0%/.2% TBST, respectively, and imaged using a LiCor Odyssey Fc.

### ChIP SEQ

For ChIP-Seq, 100 μg human cell line chromatin was immunoprecipitated with 5 μg mouse anti-GR antibody. ChIP-Seq libraries were sequenced on an Illumina High-Seq 2000. Sequence reads for each sample were mapped to the hg19 or mm9 genome assemblies as relevant with Bowtie (38). Duplicate reads were removed, and the remaining unique reads were normalized to 10 million reads. Peak calling was performed by HOMER (39) using an FDR cutoff of 0.001, a cumulative Poisson p-value of <0.0001, and required a 4-fold enrichment of normalized sequenced reads in the treatment sample over the control/input sample. Normalized sequence reads around each peak were counted in 25 bp bins. Motif discovery was conducted with HOMER package v4.2. In ChIP-Seq data we used the following settings: -size 150 –S 10 –bits (39). We limited the motif analysis to a 150 bp DNA region around each peak summit. Distance to gene and gene annotations for ChIP-Seq peaks were obtained using GREAT v1.82.

### Lentivirus

To generate lentiviral particles, 2 million Lenti-X 293T cells (Clontech, catalog #632180) were forwarded transfected with 9 μg pCMV-dR8.91, 3 μg pMD2.G and 3 μg of LentiPlasmid-of-interest (i.e. pLVX-EF1A-TET3G, pLVX-TRE3G-IRES or pTRIPZ) using FuGene6 (Promega, catalog#E2691) following the manufacturers protocol (40). After 12 hours, media was changed and viral supernatant was collected every day for 3 days and filtered through a.45micron syringe filter (Corning, catalog #431220). For each infection in each cell line, viral supernatant was titrated onto cells and mixed with 6 μg/mL polybrene (Santa Cruz, catalog# sc-134220) and incubated with cells overnight. Only cells receiving concentrations of viral supernatant that resulted in low infectivity (i.e. > 50% of the cells did not survive selection) were used to control for multiple integrations.

### Inverted invasion assays

Experiments were performed according to (41). Invasion assays were performed in 96-well dishes (PerkinElmer, Waltham, MA). In brief, cells were suspended in 2.3 mg/ml serum-free liquid bovine collagen I (Advanced Biomatrix, San Diego, CA) at 5 × 104 cells/ml, and 100-μl aliquots were dispensed into the plates. Plates were centrifuged at 1000 rpm and incubated in a 37°C/5% CO2 tissue-culture incubator. After collagen polymerization, 30 μl of medium containing 5% fetal calf serum was added on top of the collagen plug. After 36 h, cells were fixed with 4% formaldehyde (final concentration) and stained with 2 μg/ml Hoechst-33342 (Invitrogen). For dexamethasone treatment, drug was added directly to the collagen and to the medium at a final concentration of 100 nM. For quantification, 25 adjacent images were acquired in each well, yielding a total of ∼5 × 103 cells imaged per well. Nuclei labeled with Hoechst from 0 μm (bottom of the plate) to 150 μm into the collagen plug, with a 50-μm step, were detected with the object counts feature of Nikon Elements or with custom Matlab software. Invasion ratio was calculated as the sum of cell counts at 50, 100, and 150 μm over cell counts at 0 μm. Results were obtained from at least three independent experiments including five replicates on each day. Bar charts are plotted as mean of all experiments ± SEM.

### Cell line xenografts

6-8 week old female NOD/SCID mice (UTSW breeding core) were used for all *in vivo* studies. For xenograft studies, 1 x 10E6 H1993 and H2009 cells were injected subcutaneously into the right flank of female SCID-NOD mice weighing 20–25 g obtained from the University of Texas Southwestern institutional breeding core. Tumor masses were measured twice per week and mice were stratified so that tumor volumes in each group were not statistically different. Three weeks after implantation, 2 mg/kg of Dexamethasone was administered intraperitoneally (i.p.) 4 times per week (Monday through Thursday) for 4 weeks and 5mg/kg of Cisplatin was administered intravenously (i.v.) once per week (Friday) with a total of two treatments. For the combination treatments, both drugs were administered either i.p. or i.v. on the same day and same dose as single treatments and saline treatment was used as control. All mice were sacrificed when tumor burdens reached approximately 2000 mm3. Survival and tumor volume data were graphed from two separate studies, with 15 combined mice per group.

### Orthotopic/metastatic xenograft models

Orthotopic/metastatic xenograft models were generated by using 1.5 x 10E6 of Luciferase tagged A549 cells through tail vein (i.v.) injection. The orthotopic tumor volumes were evaluated using a bioluminescent imager (Xenogen Vivovision IVIS Lumina) and reported as means ± SE. Dexamethasone treatment were performed at day 15 after tail vein injection of Luciferase tagged A549 cells. Four mice were treated 2mg/kg of Dexamethasone intraperitoneally (i.p.) 4 times per week (Monday through Thursday) for 5 weeks and three mice were treated saline on the same time as control. All mice were sacrificed when control mice lost 20% of their initial body weight.

### Patient derived xenograft model

For patient derived xenograft (PDX) model, 2-3mm3 of LTL-657 PDX tumor fragments were implanted subcutaneously into the right flank of mice through surgical procedures. After the growth of tumor reached a volume around 200 mm3, 2mg/kg of Dexamethasone was administered intraperitoneally (i.p.) 4 times per week (Monday through Thursday) for 4 weeks and saline was administered on the same time as control. Tumor masses were measured twice per week and tumor volume was calculated as (0.5 x length x width2). All mice were sacrificed when tumor burdens reached approximately 2000 mm3 and tumor tissues were removed for histological examination. All animal studies were carried out under a University of Texas at Southwestern Medical Center Institutional Animal Care and Use Committee approved protocol and in accordance with the guidelines for ethical conduct in the care and use of animals in research.

### Lkb1/Kras/p53 GEMMs

Mouse strains were described previously (42). Mice were dosed with 2mg/kg of dexamethasone as described previously four times a week *via* intraperitoneal injections. MRI quantification was performed as described previously (43).

## Data availability statement

The datasets presented in this study can be found in online repositories. The names of the repository/repositories and accession number(s) can be found below: https://www.ncbi.nlm.nih.gov/gap/, phs001823.v1.

## Ethics statement

The animal study was reviewed and approved by University of Texas Southwestern Medical Center Animal Resource Center.

## Author contributions

KH- primary work and authorship. LL - Mouse work. RC- HTS screen, cell culture. HP- mouse work. KA- mouse work, western blots. SW- High throughput screening. JS- metabolic assays. RKo- ChIP seq data analysis. JF- IHC. PV- IHC. YW- PDX creation. SL- PDX creation. BT- PDX work. LG – RNAseq. All authors contributed to the article and approved the submitted version.

## Supplementary material

Supplementary Figure 1 Expression of nuclear receptors (NRs) in lung cancer cell lines. **(A)** Heatmap of RNAseq for 48 human NRs across 492 lung cancer and immortalized lung epithelial samples, including 150 NSCLC lines, 154 NSCLC PDXs, 94 SCLC cell lines, 10 NE-NSCLC lines, 21 SCLC cell line xenografts and 63 non-transformed lung epithelial cells (HBECs). **(B)** Gene Effect scores (red/green) derived from CRISPR knockout screens published by Broad’s Achilles and Sanger’s SCORE projects. Negative scores imply cell growth inhibition and/or death following gene knockout. Scores are normalized such that nonessential genes have a median score of 0 and independently identified common essentials have a median score of -1.

Supplementary Figure 2 DEX causes growth inhibition in 3-D spheroid cell culture and is dependent on the GR protein. **(A)** Images of DEX resistant cell lines (NCI-H2009, NCI-H1792) and DEX sensitive cell lines (NCI-H1993, A549) in spheroid culture plates. Cells are plated, imaged at day 2 for control and then split into two test groups. After 6 days, images of control cells and DEX treated cells are shown. **(B)** Graph showing quantitation of Cell titer glo assays with sensitive and resistant cell lines. **(C)** CRISPR clones from DEX sensitive NCI-H1993 cell lines confirming knockout of GR in immunoblots. Three clones (GR1-7, GR2-5 and GR2-7) used in subsequent assays shown in red. **(D)** Cell counting assays show loss of DEX response in DEX sensitive NCI-H1993 clones 5-days after loss of GR. Parental, DEX sensitive NCI-H1993 cell line shown in blue on graphs. CRISPR knockout clones for GR shown in red. Technical replicates for GR1-7 clone shown (a, b). **(E)**. Colony formation assays confirm loss of DEX response after loss of GR. **(F)** MTS assay confirms loss of GR does not alter response to cisplatin in 5-day assay with an IC50 value of 7.6 micromolar for NCI-H1993 and an IC50 value of 7.8 micromolar for NCI-H1993-GR2-7 GR knockout. Each cell line (H1993 and H1993 – GR2-7) was assayed with two biological replicates **(a, b)** with six technical replicates for each biological replicate. The IC50 value was calculated as an average of the two biological replicates **(a, b)**. **(G)** Radiation response assay shows no change in response due to loss of GR. Graph shows the results of 6 assays for each cell line. **(H)** Colony formation assay (14 days) with indicated platinum concentrations for parental and GR knockout line shows no change in response caused by loss of GR.

Supplementary Figure 3 DEX mediated lung cancer cell line inhibition causes morphologic changes not related to EMT and does so independently of CPS1. **(A)** Microscope images of DEX sensitive cell line (A549) show significant morphology changes and uptake of X-gal in response to DEX exposure. Images of DEX treated sensitive NCI-H1993 are shown in **(B)**. DEX treated cells show reduced collagen invasion activity, regardless of whether drug exposure occurs only during the assay (grey bars) or in cells pre-treated with DEX (black bars). **(C)** CRISPR knockdown of CPS1 does not alter DEX mediated growth arrest. Western blot confirming expression of CPS1 in parental A549 and loss of expression in CRISPR clone #2 of A549. **(D)** Cell count assay showing loss of CPS1 causes no change in DEX response phenotype in sensitive A549 cell line.

Supplementary Figure 4 DEX treatment of responder (NCI-H1993) and non-responder (NCI-H2009) cell lines does not significantly alter radiotherapy response during *in vitro* colony formation assays

Supplementary Figure 5 Examples of H&E immunohistochemistry from whole lung from the A549 metastatic model confirming significantly higher tumor burden in vehicle control treated mice when compared to DEX treated mice.

Supplementary Figure 6 Exogenous expression of CDKN1C in DEX-resistant cell lines causes cell cycle arrest. **(A)** We introduced a doxycycline (DOXY) inducible expression vector and control empty vector into two DEX-resistant lung cancer cell lines (NCI-H2009, NCI-H1299) and screened for CDKN1C expression by western blot after clonal selection. **(B)** Colony formation assay using single cell clones of DEX resistant cell lines (NCI-H1299, NCI-H2009) carrying the CDKN1C expression vector or empty vector control after doxycycline induction. **(C)** Introduction of doxycycline inducible CDKN1C expression vector into SCLC cell lines NCI-H2081 and NCI-H446 confirmed by western blot. **(D)** Colony formation assay confirming growth inhibition after doxycycline induction.

Supplementary Table 1 High throughput drug screen using 110 nuclear receptor ligands. The NR targets of each drug and CAS numbers are included.

Supplementary Table 2 CHPiseq data from DEX sensitive and DEX resistant NSCLC cell lines.
