# Supplementary figures and images for: Glucocorticoid mediated inhibition of LKB1 mutant non-small cell lung cancers
Source: Front Oncol. 2023 Mar 23;13:1025443. doi: 10.3389/fonc.2023.1025443 (PMC10078807; doi:10.3389/fonc.2023.1025443)

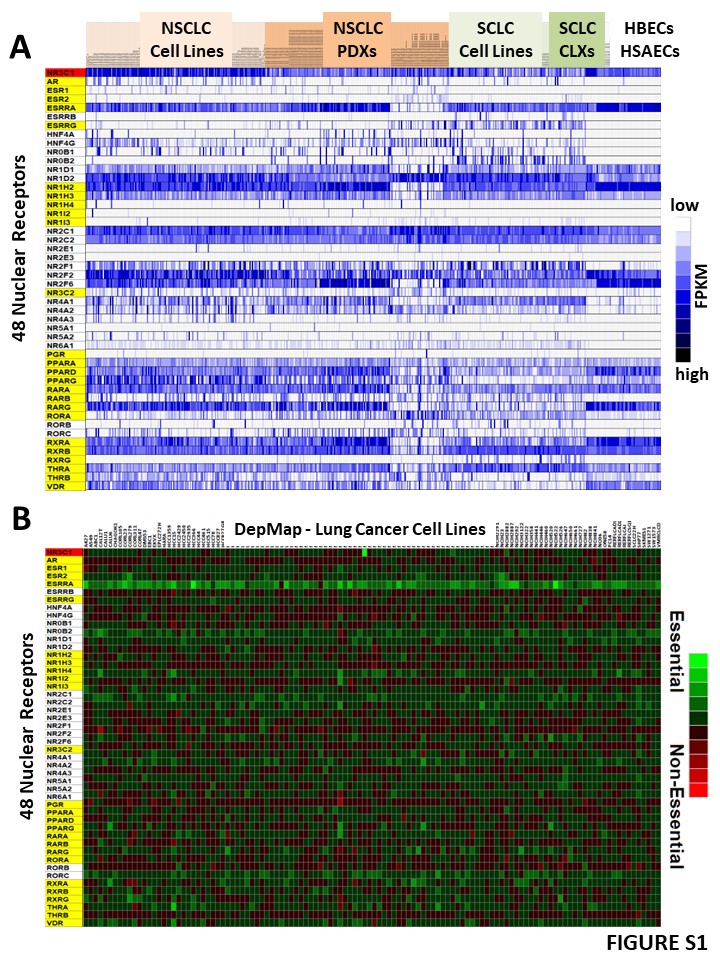

Supplement: Supplementary Figure 1 — Expression of nuclear receptors (NRs) in lung cancer cell lines. (A) Heatmap of RNAseq for 48 human NRs across 492 lung cancer and immortalized lung epithelial samples, including 150 NSCLC lines, 154 NSCLC PDXs, 94 SCLC cell lines, 10 NE-NSCLC lines, 21 SCLC cell line xenografts and 63 non-transformed lung epithelial cells (HBECs). (B) Gene Effect scores (red/green) derived from CRISPR knockout screens published by Broad’s Achilles and Sanger’s SCORE projects. Negative scores imply cell growth inhibition and/or death following gene knockout. Scores are normalized such that nonessential genes have a median score of 0 and independently identified common essentials have a median score of -1. [file Image_1.jpeg]

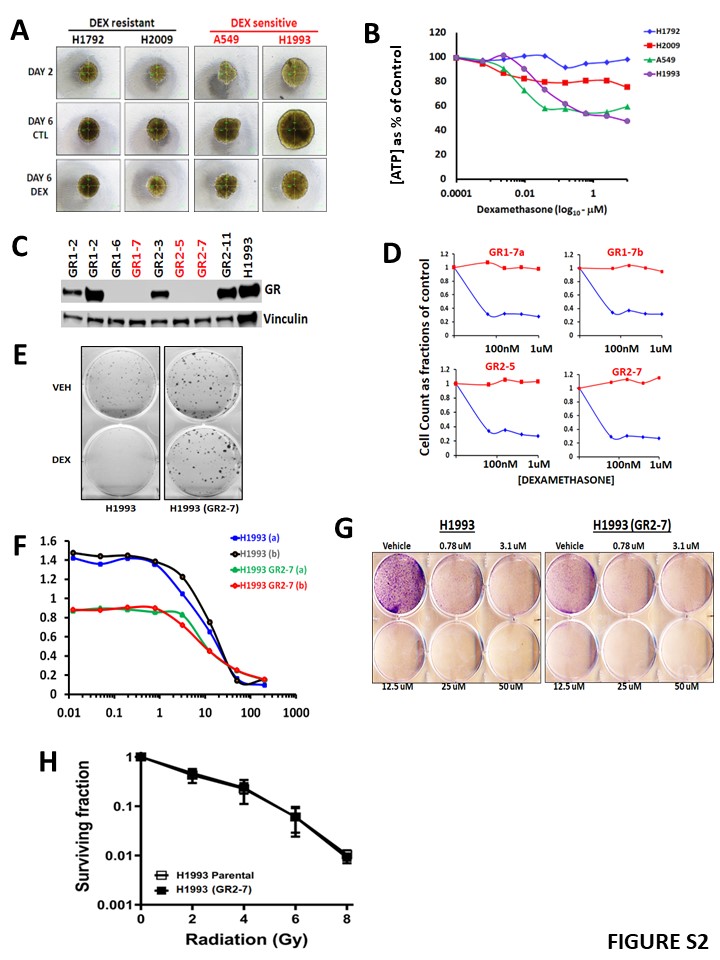

Supplement: Supplementary Figure 2 — DEX causes growth inhibition in 3-D spheroid cell culture and is dependent on the GR protein. (A) Images of DEX resistant cell lines (NCI-H2009, NCI-H1792) and DEX sensitive cell lines (NCI-H1993, A549) in spheroid culture plates. Cells are plated, imaged at day 2 for control and then split into two test groups. After 6 days, images of control cells and DEX treated cells are shown. (B) Graph showing quantitation of Cell titer glo assays with sensitive and resistant cell lines. (C) CRISPR clones from DEX sensitive NCI-H1993 cell lines confirming knockout of GR in immunoblots. Three clones (GR1-7, GR2-5 and GR2-7) used in subsequent assays shown in red. (D) Cell counting assays show loss of DEX response in DEX sensitive NCI-H1993 clones 5-days after loss of GR. Parental, DEX sensitive NCI-H1993 cell line shown in blue on graphs. CRISPR knockout clones for GR shown in red. Technical replicates for GR1-7 clone shown (a, b). (E). Colony formation assays confirm loss of DEX response after loss of GR. (F) MTS assay confirms loss of GR does not alter response to cisplatin in 5-day assay with an IC50 value of 7.6 micromolar for NCI-H1993 and an IC50 value of 7.8 micromolar for NCI-H1993-GR2-7 GR knockout. Each cell line (H1993 and H1993 – GR2-7) was assayed with two biological replicates (a, b) with six technical replicates for each biological replicate. The IC50 value was calculated as an average of the two biological replicates (a, b). (G) Radiation response assay shows no change in response due to loss of GR. Graph shows the results of 6 assays for each cell line. (H) Colony formation assay (14 days) with indicated platinum concentrations for parental and GR knockout line shows no change in response caused by loss of GR. [file Image_2.jpeg]

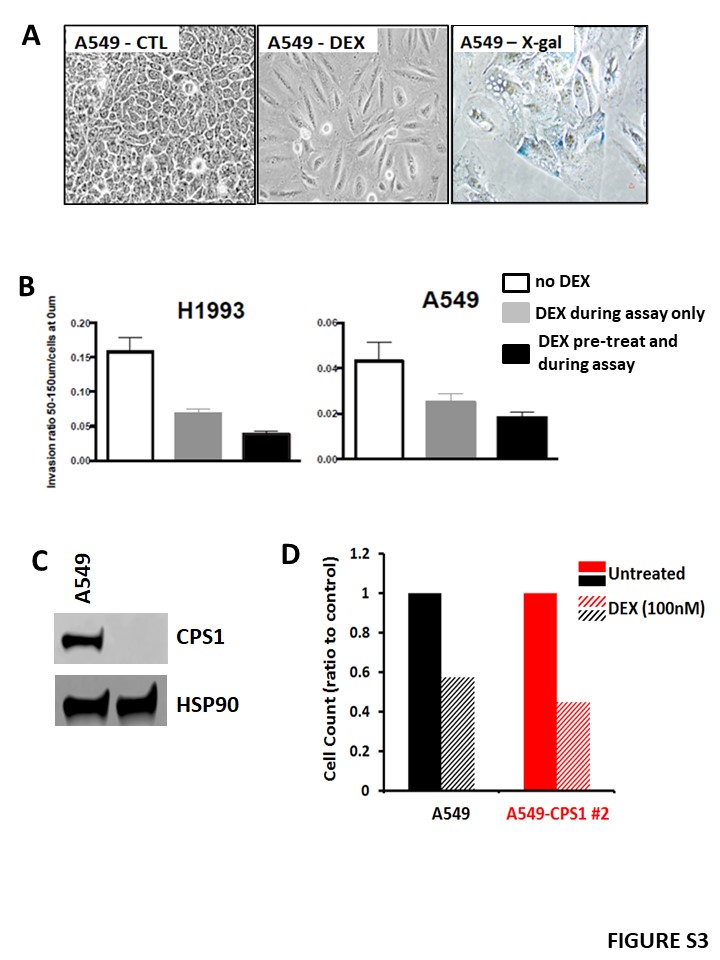

Supplement: Supplementary Figure 3 — DEX mediated lung cancer cell line inhibition causes morphologic changes not related to EMT and does so independently of CPS1. (A) Microscope images of DEX sensitive cell line (A549) show significant morphology changes and uptake of X-gal in response to DEX exposure. Images of DEX treated sensitive NCI-H1993 are shown in (B). DEX treated cells show reduced collagen invasion activity, regardless of whether drug exposure occurs only during the assay (grey bars) or in cells pre-treated with DEX (black bars). (C) CRISPR knockdown of CPS1 does not alter DEX mediated growth arrest. Western blot confirming expression of CPS1 in parental A549 and loss of expression in CRISPR clone #2 of A549. (D) Cell count assay showing loss of CPS1 causes no change in DEX response phenotype in sensitive A549 cell line. [file Image_3.jpeg]

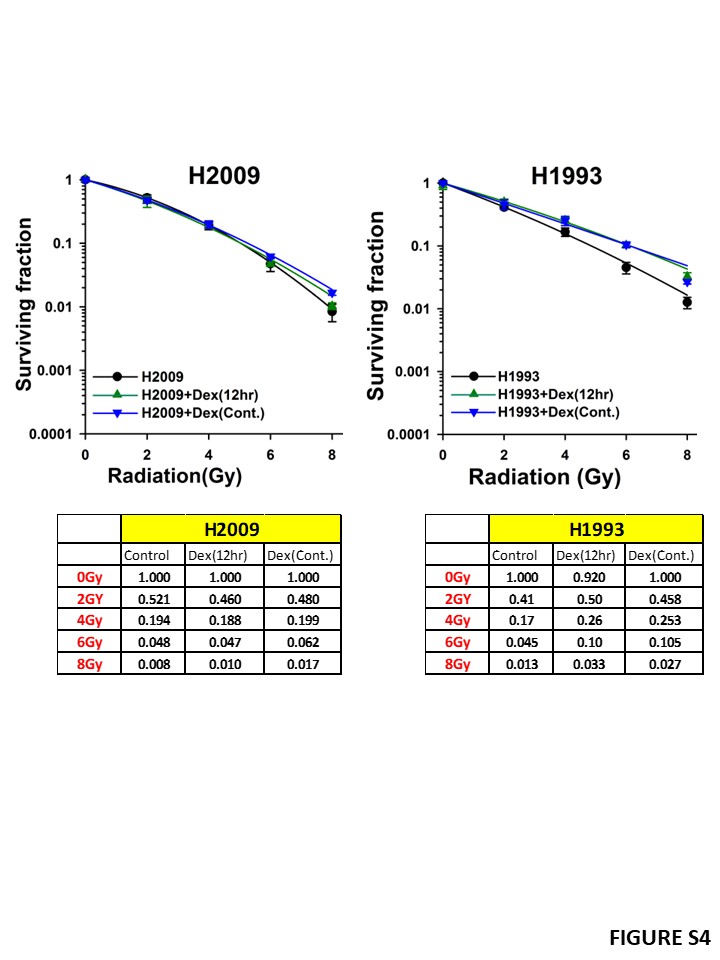

Supplement: Supplementary Figure 4 — DEX treatment of responder (NCI-H1993) and non-responder (NCI-H2009) cell lines does not significantly alter radiotherapy response during in vitro colony formation assays [file Image_4.jpeg]

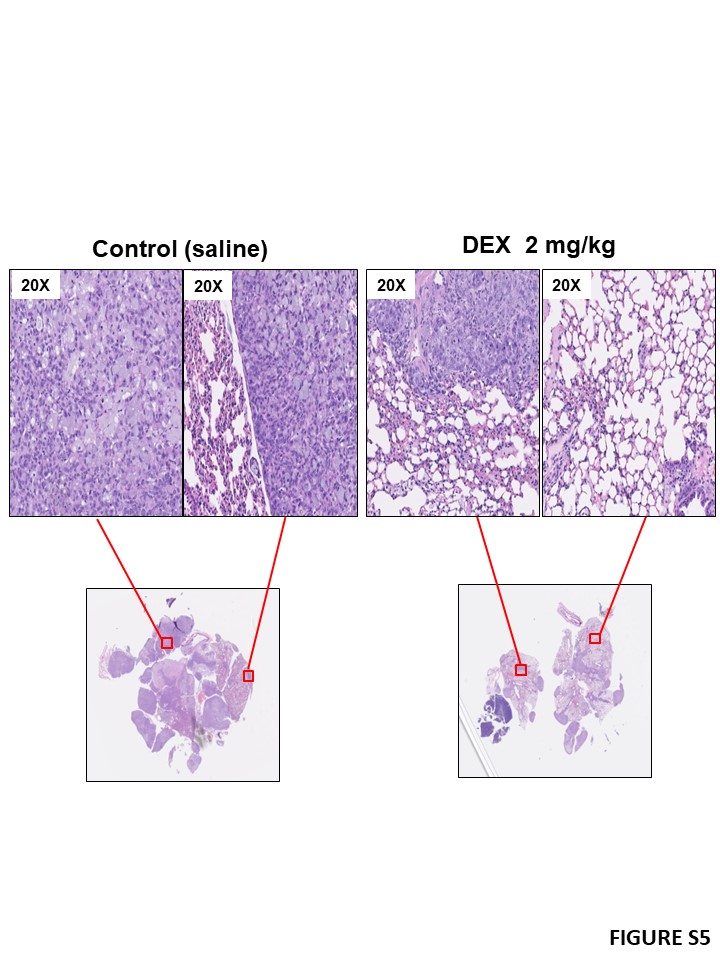

Supplement: Supplementary Figure 5 — Examples of H&E immunohistochemistry from whole lung from the A549 metastatic model confirming significantly higher tumor burden in vehicle control treated mice when compared to DEX treated mice. [file Image_5.jpeg]

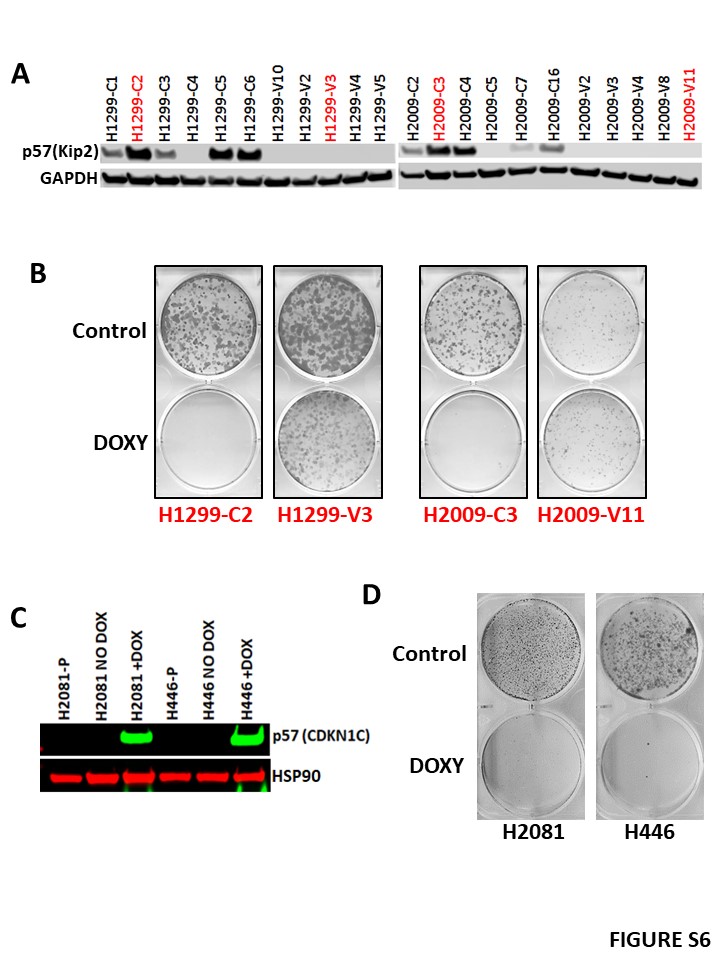

Supplement: Supplementary Figure 6 — Exogenous expression of CDKN1C in DEX-resistant cell lines causes cell cycle arrest. (A) We introduced a doxycycline (DOXY) inducible expression vector and control empty vector into two DEX-resistant lung cancer cell lines (NCI-H2009, NCI-H1299) and screened for CDKN1C expression by western blot after clonal selection. (B) Colony formation assay using single cell clones of DEX resistant cell lines (NCI-H1299, NCI-H2009) carrying the CDKN1C expression vector or empty vector control after doxycycline induction. (C) Introduction of doxycycline inducible CDKN1C expression vector into SCLC cell lines NCI-H2081 and NCI-H446 confirmed by western blot. (D) Colony formation assay confirming growth inhibition after doxycycline induction. [file Image_6.jpeg]
